# NBI cystoscopy in routine urological practice – from better vision to improve therapeutic management

**Published:** 2014-06-25

**Authors:** M Jecu, B Geavlete, R Mulţescu, F Stănescu, C Moldoveanu, L Adou, C Ene, C Bulai, P Geavlete

**Affiliations:** "Saint John" Emergency Clinical Hospital, Department of Urology, Bucharest, Romania

**Keywords:** non-muscle invasive bladder tumors, narrow band imaging, standard cystoscopy, monopolar transurethral resection

## Abstract

Abstract

Objectives: A single centre, retrospective trial was performed trying to assess the impact of NBI cystoscopy in cases of non-muscle invasive bladder tumors (NMIBT) by comparison to the standard approach. Our goal was to determine the superiority of the new method in terms of detection rates and subsequent postoperative treatment changes.

Materials and Methods: A total of 320 NMIBT suspected consecutive cases were enrolled in the study. The inclusion criteria were represented by hematuria, positive urinary cytology and/or ultrasound suspicion of bladder tumors. All patients underwent WLC and NBI cystoscopy. Standard transurethral resection of bladder tumors (TURBT) was performed for all lesions visible in WL and NBI guided resection for solely NBI observed tumors.

Results: The overall NMIBT and CIS patients’ detection rates were significantly improved for the NBI evaluation ((94.9% versus 88.1% and 95.7% versus 65.2%). Also, on a lesions’ related basis, NBI cystoscopy emphasized a significantly superior diagnostic accuracy concerning the CIS, pTa and overall NMIBT formations ((95.2% versus 60.3%, 92.8% versus 83.9% and 94.1% versus 82%). Additional tumors were diagnosed by NBI in a significantly higher proportion of CIS, pTa, pT1 and NMIBT patients (56.6% versus 8.7%, 28% versus 10.3%, 30.3% versus 10.6% and 31.6% versus 9.4%). As a result of these supplementary findings, the postoperative treatment was significantly improved in a substantial proportion of cases (15.4% versus 5.1%).

Conclusions: NBI cystoscopy represents a valuable diagnostic alternative in NMIBT patients, with significant improvement of tumor visual accuracy as well as detection rates. This approach provided a substantial amelioration to the risk category stratification and subsequent bladder cancer therapeutic management.

## Introduction

Non-muscle-invasive bladder tumors (NMIBT) represents a pathology often present in the form of a multifocal disease with a high recurrence rate within the first 5 years after the initial diagnosis [**1**]. In light of the alarmingly high recurrence rates, it became obvious that there is room for improvement in bladder cancer endoscopic diagnostic and treatment. During the past decade, the hexaminolevulinate blue light florescence cystoscopy and resection were emphasized as efficient methods in ameliorating bladder cancer detection and medium-term recurrence rates [**2**]. 

 The narrow band imaging (NBI) cystoscopy was introduced as an endoscopic alternative presumably able to improve the diagnostic accuracy in early malignant lesions [3-5]. This relatively new method is based on the light output bandwidth ranging between tight wavelengths and subsequently enhancing the color contrast of urothelial lesions [**6**]. 

 In gastroenterology, the value of NBI was already demonstrated on a rather extensive scale in the fields of colonoscopy for adenoma detection [**7**] as well as endoscopy in patients with Barrett’s esophagus [**8**]. 

 NBI cystoscopy gained increasing acknowledgement according to recent studies as a viable modality to improve urothelial tumors’ detection by providing remarkable advantages concerning small or flat lesions together with a more precise delineation of the tumoral margins [9, 10]. Following this perspective, our study was intended to bring new data possibly clarifying the actual advantages of this rather new endoscopic vision modality.

## Materials and Methods

A single centre, retrospective trial was performed while aiming to assess the impact of NBI cystoscopy in NMIBT cases when compared to the standard white light approach. Our goal was to determine the eventual superiority of the new method in terms of detection rates as well as regarding the possible postoperative treatment changes. 

 A total of 320 NMIBT suspected consecutive cases (224 men and 96 women) were enrolled in the trial. All patients underwent a standard investigation protocol which included general clinical examination, blood tests, urinalysis, urinary cytology, abdominal ultrasound and intravenous pyelography or CT-scan. The inclusion criteria were represented by hematuria, positive urinary cytology and/or ultrasound suspicion of bladder tumors. 

**Fig. 1:**
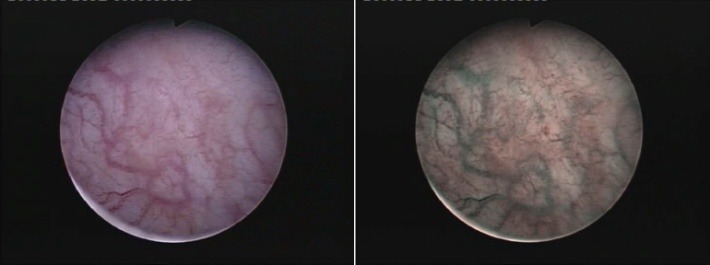
CIS lesions visible only in NBI mode

 The patients underwent WLC followed by NBI cystoscopy, resulting in separate bladder maps of all WL and respectively NBI diagnosed lesions (**Fig. 1**). Classical transurethral resection of bladder tumors (TURBT) was performed for all lesions visible at standard cystoscopy, while NBI resection was applied for lesions observed in NBI alone.

**Fig. 2:**
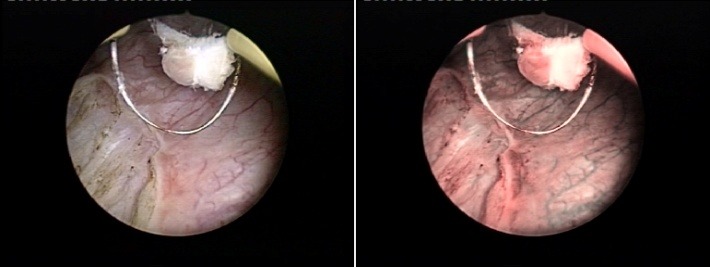
Positive tumoral margins after NBI control

 In order to control the margins of the resection areas and to identify the eventual residual lesions, a NBI control was used at the end of the procedure (**Fig. 2**). According to the pathological results, all patients without any detected tumor as well as the muscle-invasive bladder cancer cases were excluded form the trial. A second bladder map of all pathologically confirmed lesions was used in order to make a comparison to the previous ones. Subsequently, the overall NMIBT, CIS, pTa and pT1 cases’ and tumors’ detection rates were determined for both types of cystoscopy. At three months, conventional and NBI control cystoscopy was performed in all patients in order to establish the short term recurrence rate. 

## Results

All procedures were successfully carried out. A number of 22 cases (6.9%) presented no bladder cancer lesions while 298 patients (93.1%) were diagnosed with this pathology. The 45 muscle-invasive bladder cancer cases (15.1%) were excluded from the study and scheduled for radical cystectomy. A total of 253 NMIBC patients (84.9%) were pathologically confirmed. 

 The overall NMIBT and CIS patients’ detection rates were significantly improved for NBI by comparison to WLC (94.9% versus 88.1% and 95.7% versus 65.2%). Some progresses were also encountered in NBI concerning pTa cases as well (91.5% versus 86%) (**Table 1**).

**Table 1:** Patients’ detection rates

Patients’ detection rates	NBI	WLC	Patients’ no.
Overall NMIBT	240 (94.9%)	223 (88.1%)	253
CIS	22 (95.7%)	15 (65.2%)	23
pTa	150 (91.5%)	141 (86%)	164
pT1	65 (98.5%)	63 (95.4%)	66

 From the perspective of lesions’ detection rates, NBI cystoscopy emphasized a significantly superior diagnostic accuracy when compared to WLC concerning the CIS, pTa, as well as overall NMIBT formations (95.2% versus 60.3%, 92.8% versus 83.9% and 94.1% versus 82%, respectively). Additionally, more pT1 tumors were found in NBI when compared to WLC (98.3% versus 91.4%) (**Table 2**). 

**Table 2:** Tumors’ detection rates

Tumors’ detection rates	NBI	WLC	Patients’ no.
Overall NMIBC	497 (94.1%)	433 (82%)	528
CIS	60 (95.2%)	38 (60.3%)	63
pTa	323 (92.8%)	292 (83.9%)	348
pT1	115 (98.3%)	107 (91.4%)	117

 The rate of false positive results was slightly higher for NBI by comparison to standard cystoscopy (14.7% versus 12.7%). Moreover, pathologically confirmed positive tumoral margins secondary to white light TURBT were found at the NBI control in 11.4% of the cases. 

Additional tumors were diagnosed by NBI cystoscopy in a significantly higher proportion of CIS, pTa, pT1 as well as NMIBT patients in general by comparison to WLC (56.6% versus 8.7%, 28% versus 10.3%, 30.3% versus 10.6% and 31.6% versus 9.4%, respectively) (**Table 3**). 

**Table 3:** Additional tumors’ cases

Additional tumors’ cases	NBI	WLC	Patients’ no.
Global	80 (31.6%)	24 (9.4%)	253
CIS	13 (56.6%)	2 (8.7%)	23
pTa	46 (28%)	17 (10.3%)	164
pT1	20 (30.3%)	7 (10.6%)	66

 As a result of these supplementary NBI diagnostic findings, the postoperative instillation treatment was improved in a substantially higher proportion of NMIBT cases (15.4% versus 5.1%). This outcome was related to a more accurate risk category classification in light of more lesions being found in NBI. At the first cystoscopy (WL + NBI) the first cystoscopy (WL + NBI) performed at 3 months from the initial intervention, the short term recurrence rate was 7.1%. 

## Discussion

Approximately 75% of the bladder cancer patients are diagnosed with non-muscle-invasive disease, a pathology limited to the mucosa or submucosa (CIS, pTa or pT1) [**11**]. According to the literature data, between 50% and 70% of NMIBT patients display recurrence after the initial treatment [**1**]. Numerous cases of early recurrences are related to the incomplete tumor removal during TURBT [**10**]. Consequently, many methods of improving bladder cancer diagnostic and treatment were evaluated but every few actually gained a deserved place in the NMIBT diagnostic and treatment armamentarium. 

 During the recent years, it was attempted to identify alternatives to the standard diagnostic method. Fluorescence blue light cystoscopy emphasized the ability to discover more lesions, in particularly flat ones (carcinoma in situ – CIS). Many studies proved the viability of the method and showed reduced recurrence rates [**12**] as well as a more adequate postoperative instillation treatment [**13**]. 

 Due to the distinctively high costs of the photodynamic diagnosis, the search for other methods continued. Narrow band imaging, one of the most recent techniques, consists of using interference filters for the illumination of the target in the narrowed red, green and blue (R/G/B/) bands of the spectrum. This type of approach results in different images at distinct levels of the mucosa and increases the contrast between the epithelial surface and the subjacent vascular network [**14**]. 

 One of the main advantages of NBI is represented by the fact that the method has no contraindications for the patient and requires no instillation or disposable items. Furthermore, the process can be applied several times during surgery, without requiring additional costs (only related to the actual initial investment, otherwise not particularly expensive when compared to a standard endoscopic line), as it consists of a software modification to the endoscope’s optical structures [**15**]. 

 Without relying on the instillation of a chemical agent, the new technique proved its validity and lack of morbidity [**16**]. Additionally, there seemed to be no particular learning curve in diagnosing bladder tumors when using this relatively new procedure [**17**]. 

 A significantly higher overall tumor detection rate was described for NBI cystoscopy when compared to the classical WLC both in the literature (94.7% versus 79.2% [**9**]) as well as in the present trial (94.1% versus 82%). From the tumor stage approach, improved CIS (89.7% versus 50% [**10**]) and pTa (98.7% versus 82.9% [**9**]) detection rates were described for NBI cystoscopy by comparison to the standard approach according to the literature, results otherwise confirmed by our findings (95.2% versus 60.3% and 92.8% versus 83.9%, respectively). Moreover, in other papers, a significant proportion of NMIBT patients were diagnosed by NBI cystoscopy alone (12.6-26.9%) [18, 10], a perspective also demonstrated by the present trial (11.9%). 

 Regarding the overall NMIBT patients’ detection rate, a recent study showed the superiority of NBI by comparison to the standard evaluation method (97.9% and 88.8% [**19**]). From this point of view as well, the present analysis confirmed the improved accuracy of the NBI mode (94.9% versus 88.1%). 

 On the other hand, it should be mentioned NBI capacity to provide a superior delineation of tumor margins through better visualization [**10**]. Therefore, the study group identified in NBI mode extensive tumor margins surrounding large lesions that proved to remain invisible in white light, confirmed by pathology in 10.3% of the NMIBT cases. A quite similar proportion of this type of cases was determined during the course of this trial (11.4%). 

 Several studies demonstrated a significant number of additional tumors’ cases found by NBI mode (35.9-38% [6, 20, 9]). This analysis confirmed these results and viability of the method by describing a 31.6% rate for these situations.

 One of the disadvantages of NBI cystoscopy seems to consist in the high rate of false positive results when compared to WLC (31.6-36% versus 24.5-33% [**18,9**]). The figures established in the present series showed somewhat lower such rates, with no significant difference between the 2 types of cystoscopy (14.7% versus 12.7%). 

 From another perspective, it is well known that a correct postoperative treatment is crucial regarding the evolution of this disease. In these terms, the improved cystoscopyc examination under NBI resulting in more tumors found and more NMIBT cases diagnosed created the premises for a more accurate risk category determination and subsequently improved adjuvant intravesical therapy, both according to the literature (13-17% [**21,13,22**]) and to our own experience (15.4%). 

 Moreover, according to the literature, the 3 months’ short term recurrence rate in average NMIBT cases remained quite high (13.1%) [**23**]. In this regard, the respective rate displayed in this study, apparently reduced in an important proportion (7.1%), creates an optimistic perspective as to the positive oncologic impact of NBI assessment at least in the sort term evolution of NMIBT cases. Naturally, further long term confirmation in multicenter study settings involving large number of cases is required before actually acknowledging NBI cystoscopy as an integrant part of the standard bladder cancer diagnostic protocol. 

## Conclusions

NBI cystoscopy emphasized significantly improved CIS and overall NMIBT cases’ as well as lesions’ detection rates by comparison to conventional WLC. Also, substantially more pTa tumors and CIS lesions were found in NBI. The proportion of additional tumors’ cases remained important while no major difference in false positive results was found. 

 The recurrence and progression risk category determination became more accurate, thus the method providing the conditions for a superior postoperative instillation therapy to be applied. Further, along a satisfactorily reduced short term recurrence rate was emphasized at first follow-up cystoscopy, discovered a higher proportion of CIS, pTa, pT1 as well as overall NMIBT patients by comparison to WLC. 

 At the end of the day, this diagnostic alternative may very well represent effective method for identifying abnormal areas of the bladder mucosa and ultimately might gain recognition as a useful auxiliary method to standard cystoscopy. Whether NBI-assisted TUR will also result in decreased NMIBT medium and long term recurrence rates and improved disease-free survival rates remains to be clarified by the future clinical research.
